# Polymorphism in glutamate cysteine ligase catalytic subunit (GCLC) is associated with sulfamethoxazole-induced hypersensitivity in HIV/AIDS patients

**DOI:** 10.1186/1755-8794-5-32

**Published:** 2012-07-23

**Authors:** Danxin Wang, Amanda Curtis, Audrey C Papp, Susan L Koletar, Michael F Para

**Affiliations:** 1 Department of Pharmacology, Program in Pharmacogenomics, School of Biomedical Science, College of Medicine, Ohio State University, Columbus, OH, 43210, USA; 2 Division of Infectious Diseases, Internal Medicine, College of Medicine, Ohio State University, Columbus, OH, 43210, USA

**Keywords:** Idiosyncratic drug reaction, Sulfamethoxazole, Hypersensitivity, Glutamate cysteine ligase catalytic subunit (GCLC), Association, HIV/AIDS

## Abstract

**Background:**

Sulfamethoxazole (SMX) is a commonly used antibiotic for prevention of infectious diseases associated with HIV/AIDS and immune-compromised states. SMX-induced hypersensitivity is an idiosyncratic cutaneous drug reaction with genetic components. Here, we tested association of candidate genes involved in SMX bioactivation and antioxidant defense with SMX-induced hypersensitivity.

**Results:**

Seventy seven single nucleotide polymorphisms (SNPs) from 14 candidate genes were genotyped and assessed for association with SMX-induced hypersensitivity, in a cohort of 171 HIV/AIDS patients. SNP rs761142 T > G, in glutamate cysteine ligase catalytic subunit (GCLC), was significantly associated with SMX-induced hypersensitivity, with an adjusted p value of 0.045. This result was replicated in a second cohort of 249 patients (p = 0.025). In the combined cohort, heterozygous and homozygous carriers of the minor G allele were at increased risk of developing hypersensitivity (GT vs TT, odds ratio = 2.2, 95% CL 1.4-3.7, p = 0.0014; GG vs TT, odds ratio = 3.3, 95% CL 1.6 – 6.8, p = 0.0010). Each minor allele copy increased risk of developing hypersensitivity 1.9 fold (95% CL 1.4 – 2.6, p = 0.00012). Moreover, in 91 human livers and 84 B-lymphocytes samples, SNP rs761142 homozygous G allele carriers expressed significantly less GCLC mRNA than homozygous TT carriers (p < 0.05).

**Conclusions:**

rs761142 in GCLC was found to be associated with reduced GCLC mRNA expression and with SMX-induced hypersensitivity in HIV/AIDS patients. Catalyzing a critical step in glutathione biosynthesis, GCLC may play a broad role in idiosyncratic drug reactions.

## Background

Sulfamethoxazole (trimethoprim-sulfamethoxazole, TMP-SMX, cotrimoxazole) is a commonly used antibiotic against opportunistic infections associated with HIV/AIDS or other immuno-compromised states, including organ transplantation and cancer chemotherapy [1,2]. SMX-induced hypersensitivity, characterized by fever, skin rash, lymphadenopathy, and multiple organ toxicity [2], is considered an idiosyncratic adverse drug reaction with uncertain mechanisms. Such idiosyncratic adverse drug reactions common to numerous drugs (*e.g*., isoniazid, carbamazepine, phenytoin, abacavir, etc) are considered to be multifactorial and multigenic. Individual susceptibility appears to be determined by both genetic predisposition and environmental factors [3-5]. At least three distinct processes contribute: (1) production of reactive metabolites *via* drug metabolism/bioactivation; (2) reactive oxygen species (ROS) processing, and (3) binding of reactive metabolites to proteins/DNA, resulting in inflammation, cell damage, neo-antigen formation, and immune response. Polymorphisms in genes involved in all these processes may modify risk of developing idiosyncratic drug reactions.

**Figure 1:**
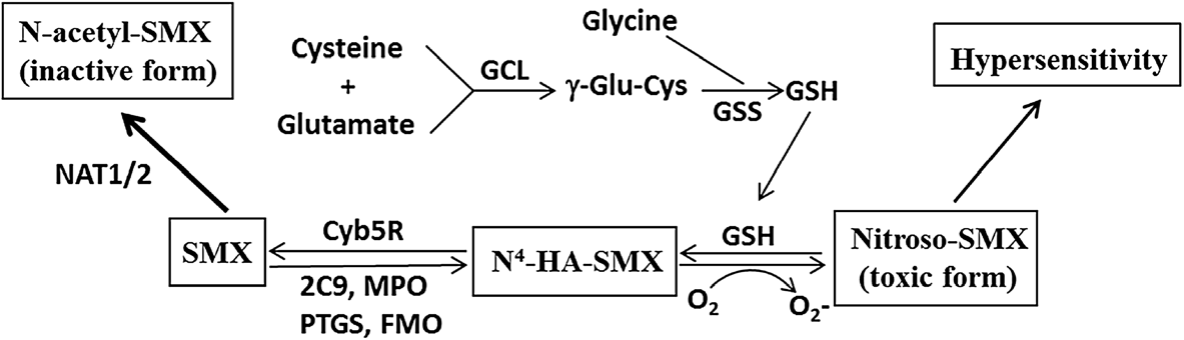
**Pathways of SMX metabolism, bio-activation and detoxification, and pathway of GSH biosynthesis.** NAT1/2, N-acetyltransferase 1 and 2; 2C9, cytochrome p450 2C9; MPO, myeloperoxidase; PTGS, prostaglandin-endoperoxide synthase; FMO, flavin containing monooxygenase; Cyb5R, NADH-cytochrome b5/cytochrome b5 reductase complex; GSH, glutathione; GCL, glutamate-cystein ligase, including catalytic and regulatory subunits GCLC and GCLM; GSS, glutathione synthetase.

SMX is predominantly inactivated through N-acetylation by two polymorphic enzymes, N-acetyltransferase 1 (NAT1) and NAT2 [6,7](Figure 1). Alternatively, SMX can be activated by cytochrome P450s (mainly CYP2C9) in the liver, or by peroxidases (MPO) [8], flavin-containing monooxygenases (FMOs) [9], and prostaglandin-endoperoxide synthase (PTGSs) [10] in liver or target tissues, producing toxic N^4-^hydroxylamine-SMX (HA-SMX). HA-SMX can auto-oxidize *via* nitroxide-SMX to nitroso-SMX [11]. This highly reactive product [6,12] binds to cellular proteins, forming neo-antigens, and triggers human major histocompatibility complex (HMC) restricted T-cell mediated immune response [13]. Nitroso-SMX can be reduced by glutathione (GSH) into HA-SMX, then HA-SMX is reduced back to SMX by NADH-cytochrome b5/cytochrome b5 reductase. Therefore, GSH is the main cellular antioxidant, scavenging reactive metabolites and preventing tissue damage (Figure 1).

Genetic association studies, including genome wide association studies, have identified genetic polymorphisms in HLA loci as strong risk factors for idiosyncratic drug reactions induced by abacavir [14], nevirapine [15], carbamazepine [16], allopurinol [17], lumiracoxib [18], flucloxacillin[19] and ximelagatran [20]. However, the involvement of HLA variants in SMX-induced hypersensitivity is unclear. Previously serological typing indicated an association between HLA-A30 B13 CW6 haplotype and SMX-induced skin toxicity [21]. Recently, one study has demonstrated weak association between HLA B*38 and SMX induced Stevens-Johnson syndrome [22], while another study failed to find association between SMX hypersensitivity and HLA-DRB1 (MHC class II) [23]. Although HLA polymorphisms appear to be the most penetrant risk factors for idiosyncratic adverse drug reactions in general, other genetic factors are likely to contribute as well, because 2% to 10% of HLA risk allele carriers do not develop idiosyncratic adverse drug reactions [19,20,24].

NAT2 slow acetylator genotype/phenotype was suggested to predispose to SMX hypersensitivity in non-HIV/AIDS individuals [25,26], while no such associations were observed in HIV/AIDS patients in several studies [27-29], possibly owing to reduced activities of liver drug metabolizing enzymes during HIV infection. Similarly, loss of function alleles *2 and *3 of *CYP2C9* decrease bio-activation of SMX, potentially protecting against adverse effects [30]. However, these *CYP2C9* alleles were not significantly associated with SMX hypersensitivity in HIV/AIDS patients [28]. Recently, we reported the gain of function alleles *10 and *11 in *NAT1* to be protective against SMX-induced hypersensitivity in HIV/AIDS patients, but this was only observed in patients who are slow acetylators for *NAT2*[31], a rare example of a gene-gene-drug interaction.

We hypothesized that additional polymorphisms in genes involved in SMX bio-activation, reactive metabolite detoxification and GSH homeostasis could modify risk of SMX-induced hypersensitivity. To test this hypothesis, we genotyped 77 tagging SNPs selected from 14 candidate genes in a cohort of HIV/AIDS patients who were taking cotrimoxazole to prevent opportunistic infections. Our results indicate that a polymorphism in glutamate cysteine ligase catalytic subunit (GCLC), the rate limiting enzyme in GSH bio-synthesis, is significantly associated with SMX-induced hypersensitivity.

## Results

Our study cohort comprises of a total of 420 HIV/AIDS patients who used cotrimoxazole (TMP-SMX) to prevent opportunistic infections, divided into two sub-cohorts according to time of enrollment (Table 1). Differences in age and distribution of sex between patients with hypersensitivity and patients without hypersensitivity were insignificant in cohort1 and the combined cohort, while small differences are present in cohort 2. Over 70% of patients were Caucasians, consistent with the HIV/AIDS population demographics in central Ohio in 1990s.

**Table 1:** Patient demographics

**Characteristics**	**All patients**	**Patients with**	**Patients without**	**P value**
		**hypersensitivity**	**hypersensitivity**	
Cohort 1				
Number (n)	171	39	132	
Sex, % male	89%	95%	89%	0.12
Age (years)	38 ± 9	38 ± 11	37 ± 8	0.55
Race, % Caucasian	74%	77%	73	0.68
Cohort 2				
Number (n)	249	63	186	
Sex, % male	89%	81%	92%	0.016
Age (years)	36 ± 8	34 ± 7	37 ± 8	0.014
Race, % Caucasian	78%	79%	77%	0.86
Cohort 1 + cohort 2				
Number (n)	420	102	318	
Sex, % male	92%	92%	92%	1
Age (years)	37 ± 8	36 ± 9	37 ± 8	0.47
Race, % Caucasian	86%	87%	86%	0.86

**Figure 2:**
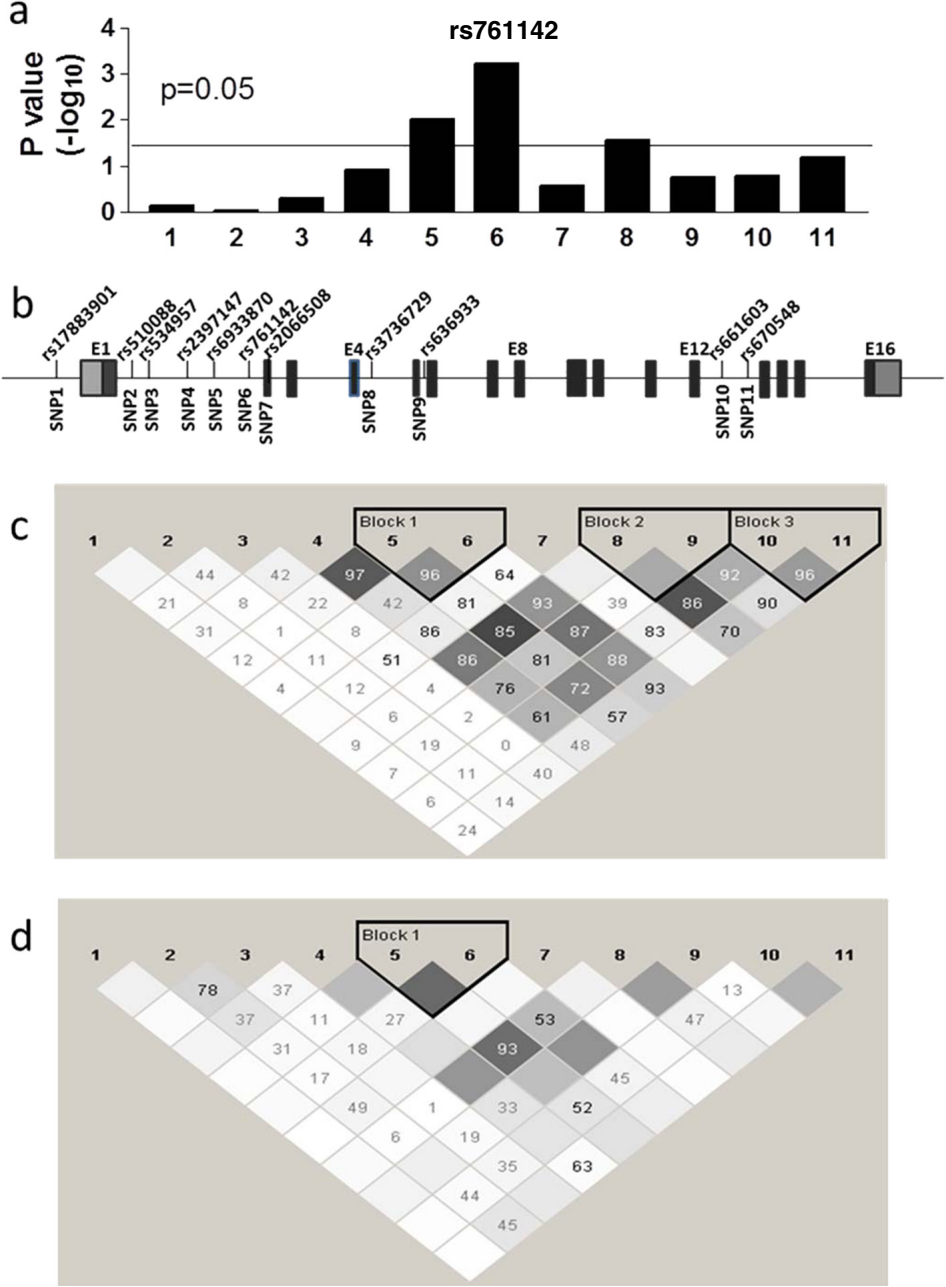
**Panel a. Association p value for SNPs in *GCLC*tested in this study.** Panel **b**. Location of SNPs inGCLC. Panel **c** and **d**. LD plots for SNPs in *GCLC* in Caucasian (panel **c**) and African American (panel **d**) populations. The numbers represent LD R.^2^

Seventy seven SNPs were successfully genotyped in samples from cohort 1 (Table 2) with call rates over 90%. The percentage of concordance is 98% for 10 duplicated samples. All SNPs followed the distribution of Hardy Weinberg’s Equilibrium with a p value >0.05. Single-SNP analysis showed 12 SNPs were significantly associated with SMX-induced hypersensitivity (basic allele test, p < 0.05) (Table 3), with a *GCLC* SNP scoring with the lowest p value (rs761142 T > G, p = 0.0006) (Figure 2). After adjusting for multiple comparisons using Bonferroni correction, rs761142 remained significant with p = 0.045.

**Table 2:** Successfully genotyped SNPs

**Gene ID**	**SNPs**
Genes involved in SMX bioactivation	
*MPO1*	No SNPs
*FMO1*	rs12720462, rs10912694, rs4916192, rs2076320, rs4433435, rs10798294
*FMO3*	rs2266782, rs1736557, rs1736560, rs3754491, rs12404218, rs2064076, rs2075992, rs7061710, rs909530
*PTGS1*	rs4273915, rs10306194, rs10306135, rs3842798
*PTGS2*	rs4648276, rs2745557, rs5275, rs5277
Genes involved in reactive oxygen species scavenging	
*SOD1*	rs202445, rs1041740, rs4998557
*SOD2*	rs2855116, rs4880, rs5746136, rs8031, rs5746092
*SOD3*	rs2536512, rs8192287, rs2695232, rs8192290
*CAT*	rs533425, rs2179625, rs554576, rs10488736, rs1049982, rs7104301
*GPX1*	rs32100, rs3811699, rs3448
*GPX3*	rs4958873, rs3792796, rs3828599, rs1946234, rs8177412, rs2070593, rs2230303, rs11548
Genes involved in GSH homeostasis	
*GCLM*	rs41303970, rs7517826, rs2301022, rs12140446, rs7549683
*GCLC*	rs3736729, rs636933, rs761142, rs6933870, rs510088, rs2397147, rs534957, rs661603, rs2066508, rs670548
*GSS*	rs2236271, rs6060127, rs2236270, rs2025096
*GSR*	rs3779647, rs2253409, rs2978663, rs2551715, rs1002149, rs8190996
*GSRG6PD*	No SNPs

**Table 3:** SNPs significantly associated with SMX-induced hypersensitivity (uncorrected p values)

**Marker**	**Gene**	**MAF**	**P value**
rs761142	GCLC	0.32	0.0006
rs2179625	CAT	0.21	0.0027
rs1736557	FMO3	0.06	0.0034
rs6933870	GCLC	0.46	0.0097
rs554576	CAT	0.46	0.013
rs2236271	GSS	0.32	0.016
rs10306135	PTGS1	0.15	0.018
rs6060127	GSS	0.28	0.018
rs7104301	CAT	0.23	0.020
rs3736729	GCLC	0.50	0.028
rs10488736	CAT	0.33	0.034
rs4958873	GPX3	0.38	0.035
rs670548	GCLC	0.25	0.065

To replicate this result, we genotyped rs761142 in DNA samples from cohort 2 and tested association with SMX-induced hypersensitivity. SNP rs761142 again showed significant association in the same direction with a p value of 0.025 (basic allele test). To further test the validity of the rs761142 association, we combining data from cohort 1 and cohort 2 and fitted the data into different genetics models. The data fitted best into an additive model, with odds ratio for TG vs TT being 2.2 (95% CL 1.4 – 3.7, p = 0.0014) and odds ratio for GG vs TT 3.3 (95% CL 1.6 – 6.8, p = 0.0010) (Table 4). Each copy of the minor G allele was associated with a 1.9 fold increase in risk (95% CL 1.4 – 2.6, p = 0.0001).

**Table 4:** Association between SNP rs761142 in *GCLC* and SMX-induced hypersensitivity

**Genotype**	**Count (%)**		**Odds ratio (95% CL)**	**P value**
	**With hypersensitivity**	**Without hypersensitivity**		
TT	31 (30%)	164 (52%)	1	
TG	54 (53%)	127 (40%)	2.2 (1.4 – 3.7)	0.0014
GG	17 (17%)	27 (8%)	3.3 (1.6 – 6.8)	0.0010

Two additional SNPs in GCLC were also significantly associated with SMX-induced hypersensitivity (Table 3, Figure 2), owing to their LD with rs761142. SNP rs670548 had been associated with GCLC expression in bronchial airway epithelial cells [32], but it did not reach significant association with SMX-induced hypersensitivity in cohort 1(Table 3 and Figure 2, P = 0.065). In the combined cohort, the association P value for rs670548 was 0.051. Because rs670548 is unevenly distributed in different populations and has very low allele frequency in African American population, we tested the association of rs670548 in Caucasians, where rs670548 was significantly associated with SMX-induced hypersensitivity (P = 0.025). However, rs761142 showed stronger association in the same cohort (P = 0.00015), indicating the association observed for rs670548 in Caucasians is a result of LD with rs761142 (D’ = 0.8 in Caucasian population, Figure 2). With the current study design (unmatched 1:3 case control ratio), and under the assumption of additive model with effect size of 2, we calculated the statistical power for cohort 1, cohort 2 and combined cohort to be 73%, 88% and 98%, respectively, to detect the effects of a polymorphism (for example rs761142) with minor allele frequency of 0.3 at α = 0.05.

Previous studies have indicated that promoter SNP rs17883901 and 5’UTR GAG trinucleotide repeats in *GCLC* are associated with schizophrenia and other diseases [33-35]. Genotyping these polymorphisms in cohort 1 showed that rs17883901 was not significantly associated with SMX-induced hypersensitivity (Additional file 1: Table S1). Similarly, none of GAG trinucleotide repeat variants showed significant associations (p = 0.32, chi-square test) (Additional file 1: Table S2). A previous study had proposed the less common genotypes (8/8, 9/9, 8/9, 7/8, ‘high risk alleles’) were associated with higher risk of developing schizophrenia compared to the more common repeats (7/7 and 7/9, ‘low risk alleles’) [35]. Moreover, red blood cells or peripheral blood mononuclear cells (PBMC) with 7/7 genotype showed changes in GCL activity and GSH levels compared to 9/9 or other genotypes [5,28]. However, we did not find an association between ‘high risk alleles’ in GCLC and SMX-induced hypersensitivity (Additional file 1: Table S1). Furthermore, promoter SNP rs17883901 and 5’UTR GAG trinucleotide repeat polymorphisms are not in linkage disequilibrium (LD) with rs761142 (Figure 2, LD D’ of 0.2 and 0.08, respectively). This result indicates that the association observed with rs761142 is unlikely to be caused by LD with previously identified promoter SNP rs17883901 or 5’UTR GAG trinucleotide repeat polymorphisms.

**Figure 3:**
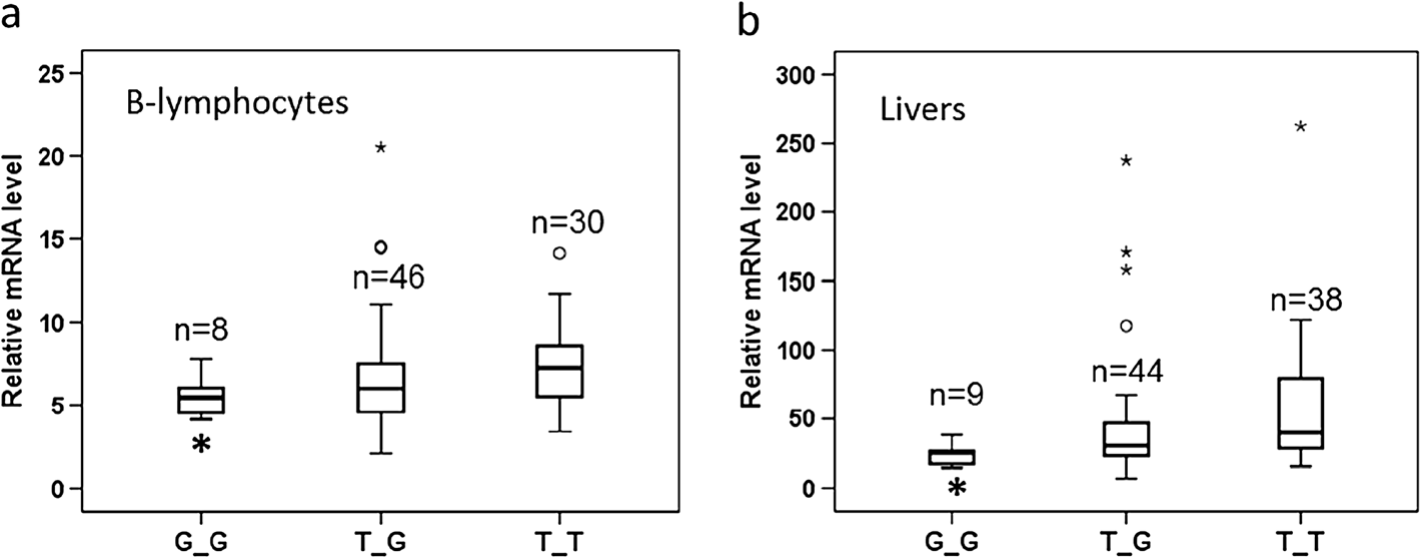
**Relative level of GCLC mRNA in B-lymphocytes (a) and livers (b) grouped by rs761142 genotypes.** *Compared to TT, p < 0.05 (t-test).

We next tested whether rs761142 affect GCLC mRNA expression in human livers and B-lymphocytes. The GCLC mRNA level was ~5% of β-actin mRNA in livers and 0.7% in B-lymphocytes. In 91 human livers and 84 B-lymphocytes, the average relative amounts of GCLG mRNA were 49 ± 5 and 7.0 ± 0.3 (mean ± SE), respectively, with considerable inter-person variability (40 fold in livers and 6 fold in B-lymphocytes). Figure 3 shows the relative GCLC mRNA levels grouped by rs761142 genotype in livers and B-lymphocytes. Samples with GG genotype showed less GCLC mRNA level than samples with TT genotype in both livers and B-lymphocytes (P < 0.05). This result indicates that the minor G allele of rs761142 is associated with reduced GCLC mRNA expression.

## Discussion

In this study, we have found rs761142 T > G in *GCLC* to be significantly associated with SMX-induced hypersensitivity in HIV/AIDS patients, with each copy of the minor G allele increasing risk nearly 2 fold. Consistent with this finding, the rs761142 G allele was also significantly associated with reduced GCLC mRNA expression in livers and B-lymphocytes. In contrast, previously reported promoter SNP rs17883901 and 5’UTR GAG trinucleotide polymorphisms [33-35] did not show significant associations. Although reactive metabolites and oxidative stress were proposed to be involved in the pathogenesis of idiosyncratic drug reactions [4,5,36], this is the first study implicating a gene involved in antioxidant defense, affecting risk of idiosyncratic drug-induced cutaneous reactions.

Glutamate-cysteine ligase (GCL), a rate limiting enzyme for biosynthesis of glutathione (GSH) (Figure 1), is composed of a catalytic subunit (GCLC) and a modifier subunit (GCLM). GSH is the main cellular antioxidant, scavenging reactive metabolites and preventing tissue damage [11,37]. In HIV/AIDS patients, GSH levels are progressively depleted [38], consistent with the higher incidence of SMX-induced hypersensitivity in HIV/AIDS patients than in non-infected controls [39]. Moreover, SMX cytotoxicity is suppressed by addition of GSH in vitro [37], and cells with GCLC knockdown were more sensitive to reactive metabolites induced cytotoxicity [40]. Given the important role of GCLC in scavenging reactive metabolites, variants that reduce GCLC expression have a plausible role in increasing risk of developing SMX-induced hypersensitivity, especially in HIV/AIDS patients with already compromised GCLC function [38].

Previous *GCLC* studies have focused on promoter SNP rs17883901 and 5’UTR GAG trinucleotide polymorphisms [33-35]. Promoter SNP rs17883901 was shown to reduce basal and H_2_O_2_-induced promoter activity [33], while the GAG trinucleotide repeat variants affect GCLC protein expression through translation [41]. However, the reported results have been inconsistent. For example, the reference 7 repeat has been associated with either lower or higher GCL activity/GSH levels compared to variant repeats (4, 8, 9 or 10 repeats) in different cell types or disease conditions [35,41-43], indicating tissue/cell or environmental specific regulation of GCLC polymorphisms, or the presence of other unidentified functional polymorphisms in *GCLC*. This is consistent with numerous conflicting clinical association studies reported for *GCLC*[33-35,44-46]. Our study failed to reveal significant association between promoter SNP rs17883901 or 5’UTR GAG trinucleotide repeat polymorphisms and SMX-induced hypersensitivity. Instead, the significantly associated rs761142 is located in the middle of intron 1 of *GCLC*. Although intronic polymorphisms can affect gene expression by various mechanisms [47], there is no evidence that rs761142 is functional by itself; instead, the association observed in this study could be caused by other functional polymorphisms in LD with rs761142 responsible for lowering GCLC mRNA expression. Similarly, SNP rs670548, located in intron12 of *GCLC* and showing significant association in our study, had also been associated with GCLC mRNA expression previously [32]. Taken together, the results indicate that a regulatory polymorphism in *GCLC* that affects mRNA expression modify risk of developing SMX-induced hypersensitivity in HIV/AIDS patients. This result warrants replication in a larger cohort. Whether the *GCLC* polymorphisms are associated with SMX-induced hypersensitivity in non-HIV/AIDS patients will require further investigation.

There are several limitations in this observational clinical association study. First, the CD4 cell counts at the time of SMX administration were not uniformly available, therefore the influence from CD4 cell count cannot be evaluated; Second, patient comorbidity and co-medication information were not available. Since SMX is inactivated and bio-activated by drug metabolizing enzymes, other disease states or concomitant administration of other drugs may affect the balance between bio-activation and bio-inactivation of SMX, influencing the level of toxic metabolites. And finally, evaluation of rs670548 and risk of hypersensitivity in African American may be limited by small sample size. A prospective larger cohort study will needed in the future to fully evaluate the association between SNPs in GCLC and SMX-induced hypersensitivity.

We have previously reported the association between polymorphisms in *NAT1* and *NAT2* and SMX-induced hypersensitivity, and gene-gene interactions between *NAT1* and *NAT2*[31]. Since idiosyncratic adverse drug reactions are thought to be multigenic, it is likely that the risks of developing hypersensitivity are modified by interactions between multiple genes. Before testing the interactions between *NAT1/NAT2* and *GCLC*, it is important to identify the functional polymorphism(s) and assess the frequency, direction and effect size for each.

Although not reported for drug-induced idiosyncratic cutaneous reaction; previous studies have associated drug induced idiosyncratic liver injury to antioxidant defense genes (SOD2 and GPX1) [48]. Consistently, SOD2 knockout mice have increased sensitivity to idiosyncratic liver injury induced by troglitazone or acetaminophen [49]. Similarly, mice deficient in NFE2L2 (NRF2), a transcription factor regulating antioxidant genes expression, also have increased sensitivity to acetaminophen induced liver injury [50]. In the present study, we observed additional SNPs in antioxidant defense genes *CATGSS* and *GPX3* to be associated with SMX-induced hypersensitivity at nominal p values less than 0.05 (Table 3). These results suggest that multiple polymorphisms in antioxidant defense genes may modify risk of developing idiosyncratic drug reaction in general.

## Conclusions

We have identified a single nucleotide polymorphism in GCLC that was significantly associated with reduced GCLC mRNA expression and with SMX-induced hypersensitivity in HIV/AIDS patients. This study supports the role of reactive metabolites and oxidative stress in the pathogenesis of SMX-induced hypersensitivity. Since oxidative stress caused by xenobiotics capable of redox cycling is a common mechanism of idiosyncratic drug reactions, it is plausible that polymorphisms in *GCLC* or other antioxidant defense genes may also be associated with idiosyncratic drug reactions caused by other drugs.

## Methods

### Patient information

Subjects included in this study had consented to an IRB-approved protocol designed to collect clinical data and specimens on HIV-infected individuals evaluated for participation in clinical trials between 1993 to 1998 in the HIV Clinical Research Unit at The Ohio State University Medical Center. A total of 420 individuals with HIV/AIDS who were taking Cotrimoxazole (trimethoprim-sulfamethoxazole) for prophylaxis or treatment of opportunistic infections and who had complete clinical data and banked blood samples available were included. This cohort was divided into two sub-groups: cohort 1, 171 patients, enrolled during 1996 to 1998 when blood was drawn using acid citrate dextrose tubes; cohort 2, 249 patients, enrolled during 1993 to 1995 when blood was drawn using heparin tubes. Since heparin was found to interfere with the SNPlex genotyping reaction, only samples from cohort1 were subjected to SNPlex genotyping. Cohort 2 served as a replication cohort with genotyping performed using other methods as described below. SMX hypersensitivity was diagnosed by presence of at least two indicator adverse drug reactions, including skin rash, fever, pruritus, etc., that disappear after drug discontinuation [44].

### Tissue samples

Human liver biopsy or autopsy samples were obtained from the Cooperative Human Tissue Network Midwestern and Western Division, under the approval of The Ohio State University Institutional Review Board. Epstein-Barr virus-transformed B-lymphocytes were obtained from Coriell Repositories. Preparation of genomics DNA, RNA and cDNA from tissues or cells was done as described [47].

### Selection of genes and polymorphisms

We selected genes based on current literatures that are involved in SMX bio-activation, reactive oxygen species scavenging and GSH homeostasis (Table 2 and Figure 1). For each gene, we selected tagging SNPs from HapMap project using the criteria of: MAF >10%, R^2^ = 80% in Caucasian population (>70% of the patients are Caucasians). Sixteen genes were initially selected; two genes (MPO1 and G6PD) did not yield any SNPs that can be successfully genotyped using SNPlex genotyping method and were excluded.

### SNPlex probe design and reagents

The select SNPs were submitted to Applied Biosystems (Foster City, California, USA) for the design of SNPlex panels following their proprietary selection algorithms. SNPlex panels and reagents were provided by Applied Biosystems as we have described previously [51].

### SNPlex genotyping

SNPlex genotyping was carried out according to the manufacture’s protocol as described in [51].

### Genomic DNA preparation

Preparation of genomic DNA from blood was performed as described [43,45].

### Other genotyping methods

GCLC 5’UTR GAG trinucleotide polymorphism was genotyped by PCR using fluorescently labeled primers (FAM labeled forward primer: GGCTGAGTGTCCGTCTCG; reverse primer (unlabeled): GAACGTCCTTGTGCCGG) followed by capillary electrophoresis separation (ABI 3730 DNA analyzer, Applied Biosystems, Foster City, California, USA)) as described [52]. Promoter SNP rs17883901 was genotyped using PCR-based restriction fragment length polymorphism methods as described [33] with modification. Instead of running agarose gels to separate and visualize the products, we labeled forward primer with fluorescent dye (FAM), and separated fragments using ABI 3730 DNA analyzer after PCR amplification and restriction enzyme digestion. SNP rs761142 was genotyped using allele specific PCR (common forward primer: CAACAGTTGGTTCTAGCAAAAGGA; reverse primer for reference allele: CCACACTGCTGGCTCTCTTGTAA; reverse for variant allele: CCACACTGCTGGCTCTCTTGTAC) as described [47].

### Quantitative mRNA analysis by real-time PCR

GCLC total mRNA levels in cDNA samples were determined by real-time PCR on an ABI 7500 sequence detection system with power SYBR Green PCR Master mix (life Technologoes). GCLC expression levels, in arbitrary units, were calculated by subtracting the β-actin cycle threshold (Ct) from the GCLC Ct to get ΔCt as described previously [47]. Arbitrary units for each sample = 1000*(2^-ΔCt^).

### Data analysis

HelixTree 6.4.3 (Golden Helix, Bozeman, MT) was used to test for Hardy-Weinberg equilibrium and basic allele Chi-square test for association with SMX-induced hypersensitivity. The associations between genotypes and hypersensitivity were analyzed using logistic regression model performed using SAS 9.1.3 software (SAS Institute, Cary, NC). The suitability of model fitting was judged by deviance goodness of fit statistics p-value and score test p-value, both of which should be larger than 0.05. The differences between mRNA levels were tested by t-test using GraphPad Prism software (GraphPad Software, La Jolla, CA). Data are expressed as mean ± SE.

## Abbreviations

SNP, Single nucleotide polymorphisms; GCLC, Glutamate cysteine ligase catalytic subunit; SMX, Sulfamethoxazole; LD, Linkage disequilibrium.

## Competing interests

The authors declare that they have no competing interests.

## Authors’ contributions

DW designed the study, performed the experiments, analyzed the data and wrote the manuscript. MC and ACP designed and performed SNPlex genotyping experiments. SLK and MFP designed the clinical study. All authors read and approved the final manuscript.

## Pre-publication history

The pre-publication history for this paper can be accessed here:

http://www.biomedcentral.com/1755-8794/5/32/prepub

## Supplementary Material

Additional file 1 **Table S1 and S2. Association between promoter SNP rs17883901 or 5’UTR GAG trinucleotide polymorphism in GCLC and SMX-induced hypersensitivity.** Distribution of 5’UTR GAG trinucleotide repeats in patients with or without hypersensitivity. Chi-square test P=0.319. Click here for file
