# Supplementary material for: Polymorphism in glutamate cysteine ligase catalytic subunit (GCLC) is associated with sulfamethoxazole-induced hypersensitivity in HIV/AIDS patients
Source: BMC Med Genomics. 2012 Jul 23;5:32. doi: 10.1186/1755-8794-5-32 (PMC3418550; doi:10.1186/1755-8794-5-32)
Supplement: Additional file 1 — Table S1 and S2. Association between promoter SNP rs17883901 or 5’UTR GAG trinucleotide polymorphism in GCLC and SMX-induced hypersensitivity. Distribution of 5’UTR GAG trinucleotide repeats in patients with or without hypersensitivity. Chi-square test P=0.319. [file 1755-8794-5-32-S1.doc]

Supplemental Table 1. Association between promoter SNP rs17883901 or 5’UTR GAG trinucleotide polymorphism in GCLC and SMX-induced hypersensitivity

| Genotypes | Count (%) | | Odds ratios (95% CL) | P value |
| --- | --- | --- | --- | --- |
|  | With hypersensitivity | Without hypersensitivity |  |  |
| Promoter SNP rs17883901 | | | | |
| CC | 31 (86%) | 103 (84%) | 1 (reference) |  |
| CT + TT | 5 (14%) | 20 (16%) | 0.83 (0.29 – 2.4) | 0.73 |
| 5’UTR GAG repeat polymorphism, comparing 7/7 genotype to others | | | | |
| 7/7 | 14 (36%) | 41 (31%) | 1 (reference) |  |
| others | 25 (64%) | 91 (69%) | 0.805 (0.380 – 1.705) | 0.57 |
| 5’UTR GAG repeat polymorphism, comparing high risk alleles to low risk alleles | | | | |
| Low risk (7/7+7/9) | 27 (69%) | 86 (65%) | 1 (reference) |  |
| High risk (others) | 12 (31%) | 46 (35%) | 0.831 (0.385 – 1.792) | 0.63 |

Supplemental Table 2. Distribution of 5’UTR GAG trinucleotide repeats in patients with or without hypersensitivity. Chi-square test P=0.319

| Genotypes | Count | |
| --- | --- | --- |
|  | Patients with hypersensitivity | Patients without hypersensitivity |
| 7/4 | 0 | 3 |
| 7/7 | 14 | 41 |
| 7/9 | 13 | 45 |
| 7/8 | 4 | 16 |
| 7/10 | 1 | 2 |
| 8/8 | 1 | 3 |
| 8/9 | 0 | 12 |
| 9/9 | 5 | 10 |
| 9/10 | 1 | 0 |
